# Genetic Ablation of Inositol 1,4,5-Trisphosphate Receptor Type 2 (IP_3_R2) Fails to Modify Disease Progression in a Mouse Model of Spinocerebellar Ataxia Type 3

**DOI:** 10.3390/ijms241310606

**Published:** 2023-06-25

**Authors:** Daniela Cunha-Garcia, Daniela Monteiro-Fernandes, Joana Sofia Correia, Andreia Neves-Carvalho, Ana Catarina Vilaça-Ferreira, Sónia Guerra-Gomes, João Filipe Viana, João Filipe Oliveira, Andreia Teixeira-Castro, Patrícia Maciel, Sara Duarte-Silva

**Affiliations:** 1 Life and Health Sciences Research Institute (ICVS), School of Medicine, University of Minho, 4710-057 Braga, Portugal; id9961@alunos.uminho.pt (D.C.-G.); id8942@alunos.uminho.pt (D.M.-F.); id8212@alunos.uminho.pt (J.S.C.); andreiacarvalho@med.uminho.pt (A.N.-C.); pg40729@alunos.uminho.pt (A.C.V.-F.); id5942@alunos.uminho.pt (S.G.-G.); id9532@alunos.uminho.pt (J.F.V.); joaooliveira@med.uminho.pt (J.F.O.); accastro@med.uminho.pt (A.T.-C.); 2 ICVS/3B’s—PT Government Associate Laboratory, 4710-057 Braga/4805-017 Guimarães, Portugal; 3 IPCA-EST-2Ai, Polytechnic Institute of Cávado and Ave, Applied Artificial Intelligence Laboratory, Campus of IPCA, 4750-810 Barcelos, Portugal

**Keywords:** astrocyte, Machado–Joseph disease, CMVMJD135 mice, IP_3_R2 KO mice, motor behavior, spinocerebellar ataxias

## Abstract

Spinocerebellar ataxia type 3 (SCA3) is a rare neurodegenerative disease caused by an abnormal polyglutamine expansion within the ataxin-3 protein (ATXN3). This leads to neurodegeneration of specific brain and spinal cord regions, resulting in a progressive loss of motor function. Despite neuronal death, non-neuronal cells, including astrocytes, are also involved in SCA3 pathogenesis. Astrogliosis is a common pathological feature in SCA3 patients and animal models of the disease. However, the contribution of astrocytes to SCA3 is not clearly defined. Inositol 1,4,5-trisphosphate receptor type 2 (IP_3_R2) is the predominant IP_3_R in mediating astrocyte somatic calcium signals, and genetically ablation of IP_3_R2 has been widely used to study astrocyte function. Here, we aimed to investigate the relevance of IP_3_R2 in the onset and progression of SCA3. For this, we tested whether IP_3_R2 depletion and the consecutive suppression of global astrocytic calcium signalling would lead to marked changes in the behavioral phenotype of a SCA3 mouse model, the CMVMJD135 transgenic line. This was achieved by crossing IP_3_R2 null mice with the CMVMJD135 mouse model and performing a longitudinal behavioral characterization of these mice using well-established motor-related function tests. Our results demonstrate that IP_3_R2 deletion in astrocytes does not modify SCA3 progression.

## 1. Introduction

Spinocerebellar ataxia type 3 (SCA3), also known as Machado–Joseph disease, is a rare autosomal dominantly inherited neurodegenerative disease [1,2] caused by an abnormal polyglutamine expansion within the ataxin-3 (ATXN3) protein [3,4]. This genetic alteration leads to slow neuronal degeneration in specific brain regions and the spinal cord, resulting in a wide variety of clinical manifestations, mainly motor-related [5,6,7]. Despite the well-described presence of neuronal death, other non-neuronal cells are involved in SCA3 pathogenesis [5,8,9,10,11,12]. Astrogliosis is a common pathological feature in SCA3 that has also been found both in humans [5,10,11,13] and in animal models [14] of the disease, suggesting an important role of astrocytes in disease pathogenesis. Astrogliosis has been considered a reaction to neuronal damage; however, studies have shown that astrocytes are activated early in SCA3, undergoing adaptive changes at times that may precede the appearance of neuropathology [15,16]. In addition, an exploratory study of cytokines present in the blood of SCA3 patients showed that the levels of eotaxin, a cytokine secreted by activated astrocytes, were significantly lower in symptomatic SCA3 patients, while asymptomatic carriers displayed higher levels of this molecule [17]. Besides that, in SCA3 patients, reactive astrocytes are present in both spared (such as the cerebral cortex) and affected brain regions (the internal segment of the pallidum, the substantia nigra, the lateral reticular nucleus, the pontine, the pre-cerebellar nuclei, and the subthalamic nuclei [5,10,11,13]. In a mouse model of SCA3, the CMVMJD135 transgenic line [14], an increase in GFAP intensity was also found in the substantia nigra but not in the pontine nuclei, both key affected brain regions. The CMVMJD135 mouse line (hereafter referred to as Q135) was generated and characterized in our lab and has been extensively used to study SCA3 pathophysiology since it displays progressive phenotypical features and neuropathology that overlap with the clinical manifestation of SCA3 [14]. Hence, they provide a good model to study disease mechanisms in SCA3, including the involvement of astrocytes in pathogenesis.

Astrocytes are the predominant glial cell type in the central nervous system and an active participant in numerous functions in the brain, including the maintenance of homeostasis and the modulation of brain circuits [18,19,20]. Although astrocytes are not electrically excitable, several studies have shown that astrocytes can sense, integrate, and respond to neuronal activity by raising the intracellular calcium ion concentration [21]. It has also been demonstrated that astrocytic calcium elevations may occur spontaneously or via elicitation of neuronal activity [18,22,23,24]. The calcium increases are dependent not only on its entry from the extracellular space but also from its release from the intracellular storages within organelles such as mitochondria, the nucleus, and its major storing compartment—the endoplasmic reticulum (ER). While calcium from mitochondria is released via the mitochondrial permeability transition pore, calcium released from the ER occurs via ryanodine and inositol 1,4,5-trisphosphate receptors (IP_3_Rs) [25,26,27,28]. The three main isoforms of IP_3_Rs (IP_3_R1, IP_3_R2, and IP_3_R3) are encoded by three distinct genes (*ITPR1*, *ITPR2*, and *ITPR3*), differing in subcellular localization, expression pattern, and functional properties [29]. The three isoforms share about 60–70% amino acid similarity [30,31]. While IP_3_R1 is predominantly present in neuronal cells, IP_3_R2 is mostly expressed in glial cells, e.g., astrocytes, cardiomyocytes, and hepatocytes, and IP_3_R3 is present in many different cell types, e.g., epithelial cells, adult pancreatic islets cells, and gastrointestinal tract cells [32,33,34].

Aberrant calcium signalling has also been implicated in the pathogenesis of SCA3. Interestingly, it was shown that inhibition of calcium-dependent protease calpain suppressed the aggregation of the pathological ataxin-3 in cells and prevented neurodegeneration [35,36]. Also, mutant ataxin-3 was shown to physically interact with and disrupt the function of IP_3_R1, expressed in neurons, to increase calcium release and cause Purkinje cell excitability [37]. These findings are similar to the ones described previously in animal models of HD [38,39,40] as well as SCA2 [41] and provide support for the assumption that several polyglutamine-expansion disorders might share common pathogenic mechanisms. Furthermore, SCA3-YAC-84Q transgenic mice fed with dantrolene, a clinically relevant calcium signalling inhibitor, showed an improvement in motor performance and prevented neuronal loss in affected brain regions, such as the pontine nuclei and the substantia nigra [37]. Thus, these results indicate that abnormal calcium signalling may play an important role in SCA3 pathology.

Calcium-dependent astrocytic functions, such as the release of gliotransmitters, rely upon the process of calcium signalling in response to neuronal activity, predominantly by IP_3_R2-mediated release from internal stores [23,24,34]. Consequently, the IP_3_R2 KO mouse model [42], a mutant with altered astrocytic calcium signalling due to the absence of IP_3_R2, has been at the center of several relevant studies that have revealed important physiological roles of IP_3_R2 signalling in not only astrocytes [43,44,45,46,47,48,49,50,51] but also in pathophysiological alterations in the context of stroke [52,53], traumatic brain injury [54], and some neurodegenerative diseases, such as Alexander disease [55]. The disruption of astrocytic calcium signalling by genetically ablating IP_3_R2 had a beneficial effect on neuronal protection and motor deficits after stroke [56]. On the contrary, the ablation of IP_3_R2 in the context of amyotrophic lateral sclerosis was detrimental to SOD1^G93A^ mice, increasing innate immunity that contributed to the significantly shorter lifespan of these animals [57]. These contradictory results have generated controversy about the functional significance of calcium signalling by IP_3_R2 in astrocytes, since it has been assumed that calcium signalling is abolished in IP_3_R2 KO mice [43,58,59]. However, it was later demonstrated that there is calcium signalling generated by other sources in IP_3_R2 KO astrocytes [28], which may compensate to some extent for the absence of IP_3_R2.

To the best of our knowledge, IP_3_R2 has not been linked to SCA3 pathogenesis so far, despite evidence suggesting the involvement of astrocytes and the calcium signalling pathway in SCA3 pathogenesis. In the current study, we aimed to investigate the relevance of IP_3_R2 in the onset and progression of SCA3. Here, we investigated whether IP_3_R2 depletion and the consecutive suppression of global astrocytic calcium signalling would lead to marked changes in the behavioral phenotype of CMVMJD135 mice. Therefore, we crossed the CMVMJD135 mouse model [14] with the IP_3_R2 KO mouse model [42] and performed an extensive longitudinal characterization of the motor phenotype of these mice using a panel of well-established behavioral tests in order to determine the effect of IP_3_R2 ablation in the onset and disease presentation of SCA3.

## 2. Results

### 2.1. IP_3_R2 Expression in the CMVMJD135 Mouse Model

**Figure 1:**
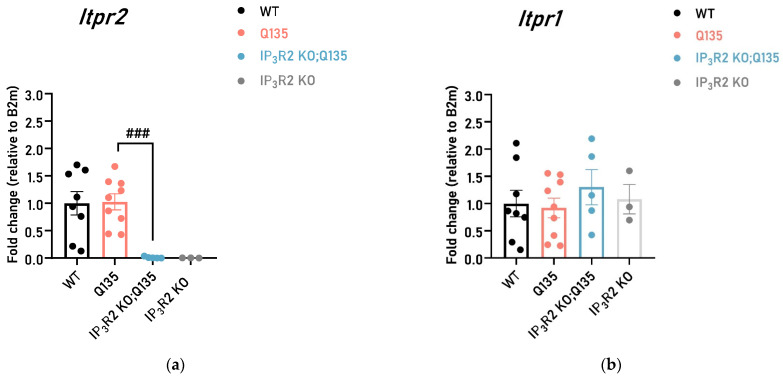
The relative expression of IP_3_ isoforms levels in the brainstem of WT, Q135 mice, and IP_3_R2 KO; Q135 mice measured by RT-qPCR. (**a**) The mRNA levels of IP_3_R2 were not detected in the IP_3_R2 KO; Q135 mice as expected; (**b**) No differences were found between the Q135 and IP_3_R2 KO; Q135 mutant mice regarding the IP_3_R1 expression levels. Relative gene expression was calculated using the 2^−ΔΔCt^ relative quantification method. One-way ANOVA with Tukey HSD or Dunnett T3 comparisons was carried out. All statistical information is included in Appendix A, Table A1. The data are presented as the mean ± SEM. Hashtags (###) indicate statistical significance between the Q135 and IP_3_R2 KO; Q135 mice. The means were considered statistically significant at a *p*-value ### *p* < 0.001.

To investigate the relevance of IP_3_R2 in SCA3, we first assessed whether *Itpr2* gene expression was altered in the Q135 mice as well as to confirm IP_3_R2 deletion in the double mutant animals (described here as IP_3_R2 KO; Q135). We showed that the mRNA levels of *Itpr2* are not altered in the brainstem of the Q135 mouse model, a well-known affected brain region in SCA3 (Figure 1a). Importantly, and as expected, IP_3_R2 is not present in the IP_3_R2 KO; Q135 animals as levels of *Itpr2* mRNA in these mice were not detected (Figure 1a). To rule out the possibility that deletion of IP_3_R2 could induce compensatory expression of other IP_3_Rs, we assessed the expression levels of the IP_3_R1 isoform. No differences were found (Figure 1b), suggesting that no adaptive compensations occurred in this constitutional KO strain, as we previously showed for the IP_3_R2 KO model [51].

### 2.2. The Double Mutant Mice Show Progressive Neurological Deficits Similar to CMVMJD135 Animals

**Figure 2:**
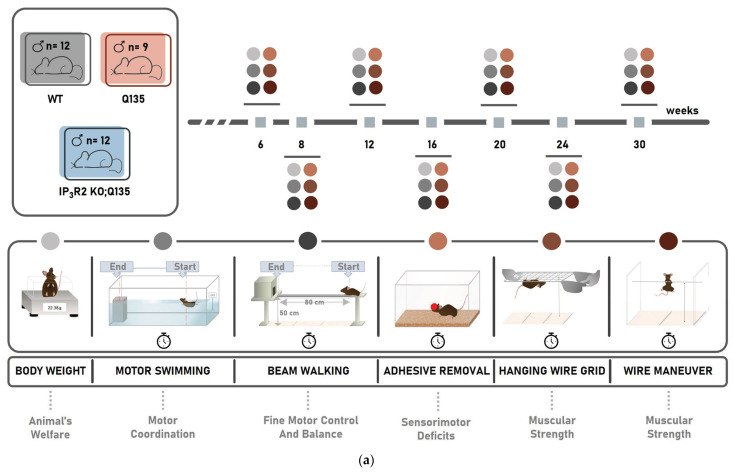

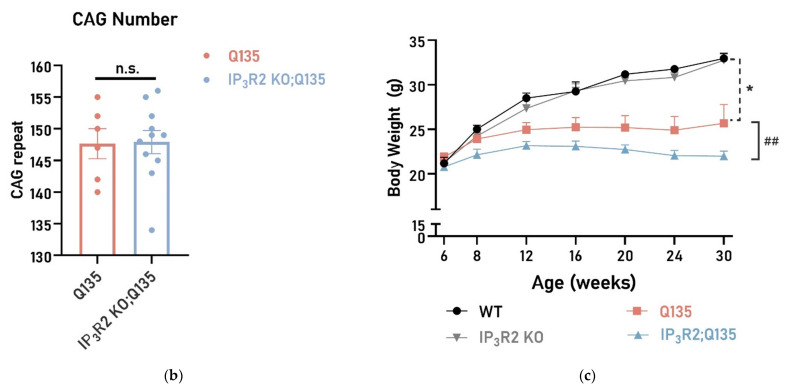
IP_3_R2 ablation does not change the motor phenotype of the Q135 mice. (**a**) Schematic representation of the study design. (**b**) CAG repeat number comparison between the testing groups. (**c**) Assessment of body weight with age. An independent *t*-test, repeated measures ANOVA, and one-way ANOVA were carried out. All statistical information is included in Appendix A, Table A1. The data are presented as the mean SEM or as a percentage of animals (%). Asterisks (*) indicate statistical significance between the WT and Q135 mice, while hashtags (##) indicate statistical significance between the Q135 and IP_3_R2 KO; Q135 mice. The means were considered statistically significant at a *p*-value * *p* < 0.05.

Next, we performed a battery of well-established motor behavioral tests to obtain a full characterization of the double mutant to determine the effect of IP_3_R2 ablation in the disease presentation. In this longitudinal behavioral characterization, the animals were tested on several behavioral paradigms at 6, 8, 12, 16, 20, 24, and 30 weeks of age (Figure 2a), corresponding to early, medium, and advanced disease stages, as previously described for Q135 mice [14]. The analysis of CAG length variation revealed that the Q135 and IP_3_R2 KO; Q135 mice carried a similar number of CAG repeats (Figure 2b), excluding an influence of CAG length variation on the behavior of the different test groups. All mice gained weight at a similar rate until 12 weeks of age (Figure 2c); however, the Q135 and IP_3_R2 KO; Q135 mice stopped gaining weight throughout the age groups, showing a significantly lower body weight gain compared with their WT-littermates at 20, 24, and 30 weeks of age. In contrast, the control animals continued to gain weight until 30 weeks of age (Figure 2c). The IP_3_R2 KO; Q135 mice displayed a lower body weight over time when compared to the Q135 mice (Figure 2c).

**Figure 3:**
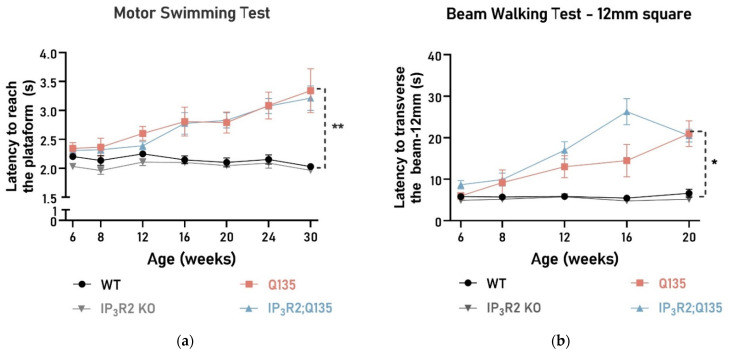

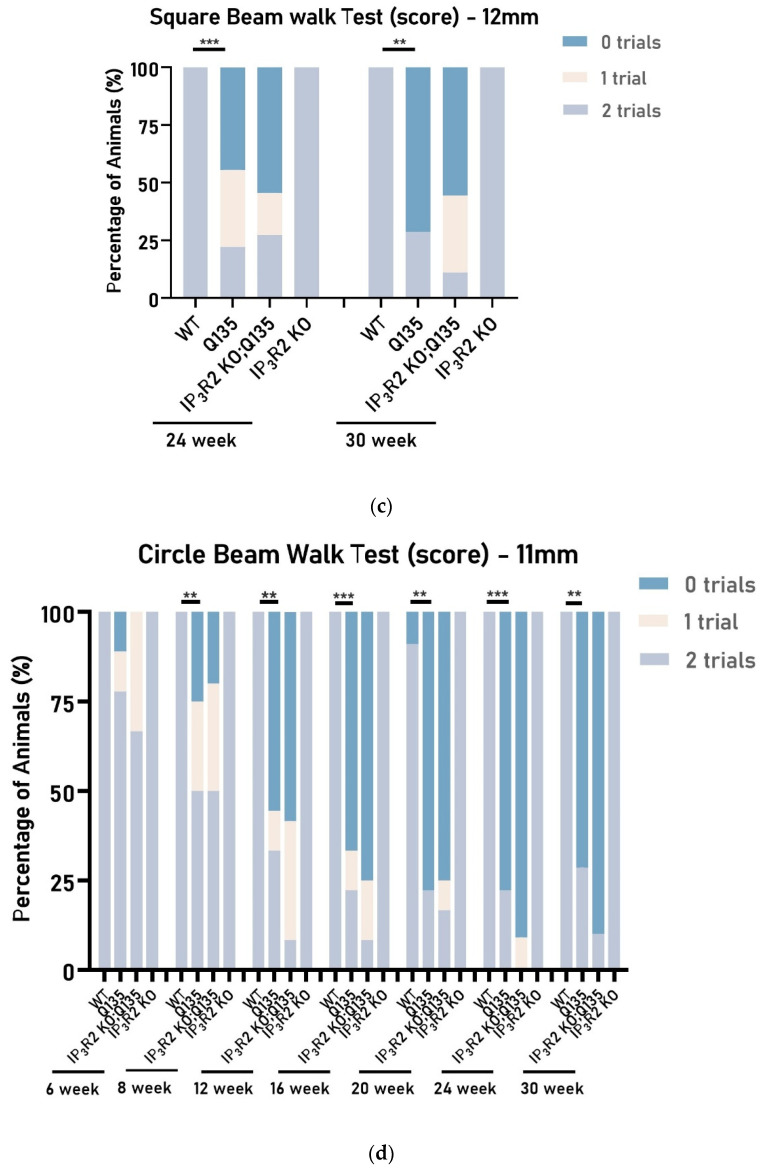
IP_3_R2 ablation does not alter the motor performance of the Q135 mice in the behavioral tests. (**a**) In the motor swimming test, the IP_3_R2 KO; Q135 animals’ performance was similar when compared to the Q135 mice. The balance beam test performance of the WT, Q135, and IP3R2 KO; Q135 mice using the 12 mm square beam (**b**,**c**) and the 11 mm circle beam (**d**). Significant differences were observed in the beam walk test between the WT and the Q135 animals. However, the IP_3_R2 KO; Q135 mice performed similarly to the Q135 mice. Repeated measures ANOVA, one-way ANOVA, and the Kruskal–Wallis test were carried out. All statistical information is included in Appendix A, Table A1. The data are presented as the mean SEM or as a percentage of animals (%). Asterisks (*) indicate statistical significance between the WT and Q135 mice. The means were considered statistically significant at a *p*-value * *p* < 0.05, ** *p* < 0.01, and *** *p* < 0.001.

In the motor swimming test, no significant differences were found between the Q135 and IP_3_R2 KO; Q135 mice in their swimming performance (Figure 3a), with their latency to traverse the water tank being significantly worse than that of the WT mice during aging. As the disease progresses, the Q135 mice have increasing difficulty in maintaining balance [14]. Both the Q135 and IP_3_R2 KO; Q135 mice showed similar performance on the balance beams (Figure 3b–d).

**Figure 4:**
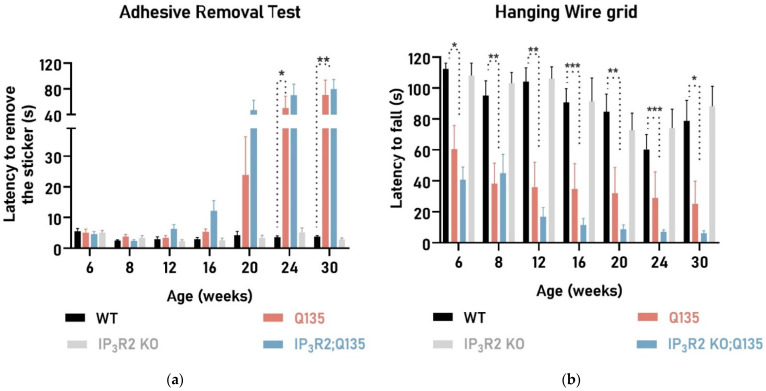

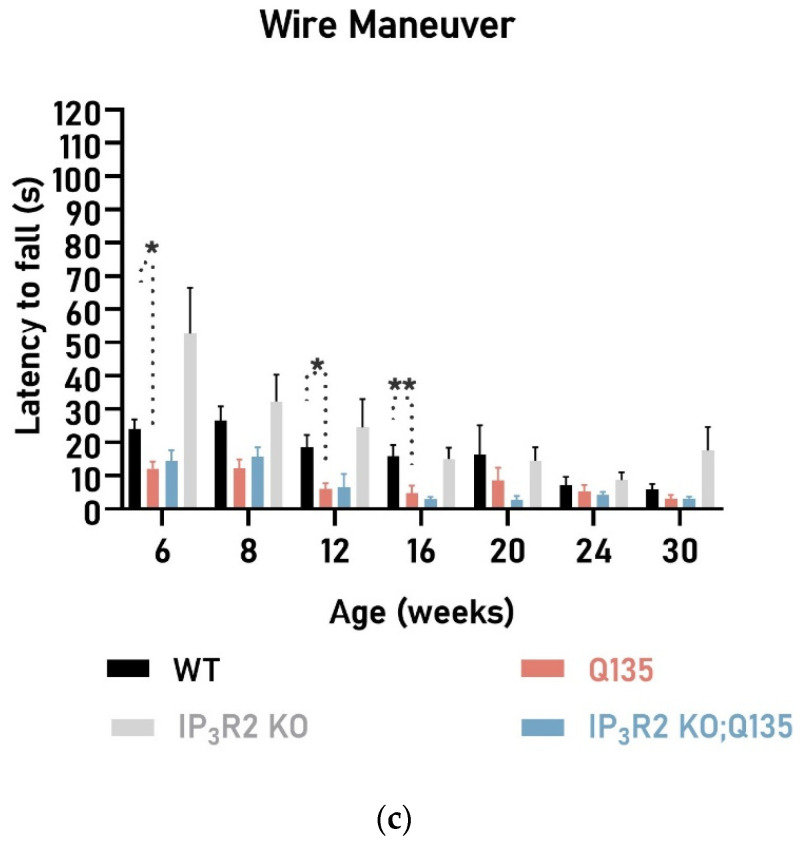
Both the Q135 and IP_3_R2 KO; Q135 animals display similarly reduced strength and slower movement initiation. (**a**) In the adhesive removal test, the IP_3_R2 KO; Q135 and Q135 mice showed worsening of their performance over time. Evaluation of limb muscular strength assessed by the (**b**) hanging wire grid and the (**c**) wire maneuver test. Assessment of limb strength showed no significant differences between the Q135 and IP_3_R2 KO; Q135 mice. Repeated measures ANOVA, one-way ANOVA, and the Kruskal–Wallis test were carried out. All statistical information is included in Appendix A, Table A1. The data are presented as the mean SEM. Asterisks (*) indicate statistical significance between the WT and Q135 mice.The means were considered statistically significant at a *p*-value * *p* < 0.05 and ** *p* < 0.01, *** *p* < 0.001.

Next, we evaluated whether the IP_3_R2 KO; Q135 mice showed any sign of movement initiation deficits, seen as a measure of parkinsonism [60,61] a clinical feature of some SCA3 patients, with the adhesive removal test (Figure 4a). Both the Q135 and IP_3_R2 KO; Q135 animals showed an increased latency to remove the nose sticker when compared to their WT-littermates, suggesting movement initiation deficits for both genotypes. Again, no differences were seen in this test between the IP_3_R2 KO; Q135 and Q135 mice (Figure 4a).

Loss of muscular strength is a very early and severe symptom observed in Q135 mice, with it already being observed when the animals were 6 weeks of age [14]. Thus, forelimb and hindlimb strength were also evaluated. In the hanging wire grid (Figure 4b), the Q135 mice showed a significantly lower latency to fall from the grid when compared to their WT-littermates, suggesting that limb muscular strength is diminished in these animals. The IP_3_R2 KO; Q135 mice presented a similar phenotype as the Q135 mice. Accordingly, both the Q135 and IP_3_R2 KO; Q135 mice displayed lower forelimb strength measured by the wire maneuver test (Figure 4c).

## 3. Discussion

In this work, we explored the contribution of IP_3_R2, the main IP_3_ receptor responsible for somatic calcium signalling in astrocytes, to the progression of motor symptoms in an animal model of SCA3. Our results show that ablation of IP_3_R2 had no major impact on the motor-related symptoms of a SCA3 animal model, as the IP_3_R2 KO; Q135 mice displayed a similar motor phenotype when compared to the CMVMJD135 mouse model.

In the brainstem of the Q135 mice, a key affected brain region, the baseline mRNA levels of *Itpr2* were not altered when compared to the WT mice. The expression levels of the homologous isoform (IP_3_R1) were also analyzed to understand whether the deletion of IP_3_R2 could affect other IP_3_Rs expression changes through a direct compensatory mechanism or through interactions between IP_3_R2 and other targets. Our results excluded such compensatory changes.

We confirmed previous observations that Q135 mice display significantly lower body weight gain compared to WT mice and a decline in body weight as the disease progresses. The decline in body weight as the disease progress is most likely associated with the significant atrophy observed in the model [14]. Interestingly, over time, the IP_3_R2 KO; Q135 animals displayed a lower body weight when compared to the Q135 mice. This suggests that IP_3_R2 can be a modulator of this more “peripheral” aspect of the phenotype. IP_3_R2 KO mice have been reported by us [51] and others [47,56] to have a similar body weight when compared to WT mice; however, in the context of SCA3, the absence of IP_3_R2 in the IP_3_R2 KO; Q135 animals appears to have induced the loss of body weight. This suggests that IP_3_R2 could have a detrimental effect on peripheral tissues, and further studies are needed to clarify the functional role of IP_3_R2 in SCA3 mice. Studies with calcium imaging and/or experiments of knock-down of IP_3_Rs in the peripheral tissues [62,63] could help us to elucidate this point and its relevance for SCA3.

Using tests to evaluate core motor deficits, such as those affecting movement, coordination and balance, our data showed that, unlike what was seen for SCA2 [41] the genetic deletion of IP_3_R2 does not alter the disease progression of Q135 animals. Similar results have been described in amyotrophic lateral sclerosis (ALS) mouse models, where the genetic ablation of IP_3_R2 showed no impact on disease onset and motor coordination although worsened muscular strength and decreased survival of the SOD1^G23A^ mice [57]. At first glance, the involvement of IP_3_R2 in ALS seems controversial, as higher levels of *Itpr2* were shown to be detrimental to the mice and therefore, it would be expected that the ablation of this isoform could have beneficial effects [64]. Contrary to our observations and those made in ALS mice, ablation of this receptor in the ischemic brain as well as in aged mouse brains was shown to be neuroprotective, while reducing behavioral deficits [56]. These observations may indicate a distinct role of astrocytes in different contexts and disease aetiologias. The physiological role of IP_3_R2-mediated calcium signalling needs to be studied in different pathological contexts since the absence of IP_3_R2 leads to different outcomes according to the context.

We also performed a battery of tests to evaluate muscular strength and movement initiation, a parkinsonism-related manifestation. In these behavioral dimensions, the performance of the IP_3_R2 KO; Q135 mice was also indistinguishable from that of the Q135 mice. Overall, these data suggest that the deletion of IP_3_R2 had no impact on the motor activity of the IP_3_R2 KO; Q135 mice. Despite the likely suppression of global calcium signalling in astrocytes, the IP_3_R2 KO mice still have IP_3_R2-independent calcium signals through alternative calcium sources and synaptic calcium, such as plasmalemma calcium influx or the mitochondria [27,28,65]. Based on this, it is plausible to question whether the transient calcium signals are robust enough to justify the lack of impact of IP_3_R2 deletion in the SCA3 background. Additionally, the possibility of a compensatory mechanism between IP_3_R2 and other critical players of calcium signalling exists, including G-protein-coupled receptors. Further studies should be performed to unravel the involvement of other calcium signalling pathways in SCA3 onset and progression, including approaches using pharmacological approaches [63,66,67,68] or the two-photon excitation microscopy technique [69,70].

Globally, our results demonstrate that SCA3-IP_3_R2 double mutants present similar behavior to SCA3 mice, suggesting that the IP_3_R2 receptor is not a major modulator of the onset and progression of SCA3 motor symptomatology.

## 4. Materials and Methods

### 4.1. Animal Generation and Maintenance

In this study, two mouse models (Mus musculus and strain C57BL/6J) were used to understand the potential involvement of IP_3_R2 receptor deletion in SCA3 progression.

The Q135 mice were generated as previously described [14]. The CMVMJD135 mouse model expresses human ATXN3 (cDNA, isoform 3c) under the control of the CMV promoter (ubiquitous expression) at near-endogenous levels [14]. The cDNA variant of the ATXN3 gene carries a repeat tract with the sequence (CAG)2CAAAAGCAGCAA(CAG)129, coding for 135 glutamine [14,71]. The IP_3_R2 knock-out (KO) mouse model was supplied by Prof. Alfonso Araque (Minneapolis, MN, USA) [72] under agreement with Prof. Ju Chen (U.C. San Diego, CA, USA) [42]. Initially, the heterozygous *Q135* mice were crossed with homozygous IP_3_R2 KO mice, and the following genotypes were obtained: wild-type (WT), heterozygous *Q135* homozygous IP_3_R2 KO (IP_3_R2 −/−), and heterozygous IP_3_R2 KO (IP_3_R2 +/−) mice, as well as a double mutant of heterozygous Q135 and heterozygous IP_3_R2 KO (IP_3_R2 +/−; *Q135*). To generate the experimental groups, the obtained double mutants (IP_3_R2 +/−; *Q135*) were crossed with the heterozygous IP_3_R2 KO (IP_3_R2 +/−), obtaining the following genotypes WT, *Q135*, IP_3_R2 −/−, IP_3_R2 +/−, and IP_3_R2 −/−; *Q135*. To answer our main question, although we performed the behavior analysis with all the obtained genotypes, we focused on the comparison between the WT, *Q135*, and IP_3_R2 −/−; *Q135* mice. Male mice were used in this study to increase analysis homogeneity (decrease variance) and to allow for a small number of animals. Because the IP_3_R2 heterozygous animals had not been characterized before, we included this group of mice in our analyses. No differences were found in any of the motor-related tests performed when compared to the WT-littermates (see the statistical analyses in Appendix A, Table A1).

The animals were maintained in a conventional animal facility and under standard laboratory conditions, which included an artificial 12 h light/dark cycle, lights on from 8:00 am to 8:00 pm, an ambient temperature of 21 ± 1 °C, and a relative humidity of 50–60%. The mice were given a standard 4RF25 diet during the gestation and postnatal periods and a 4RF21 diet after weaning (3 weeks of age; Mucedola SRL, Settimo Milanese, Italia) as well as water ad libitum. During weaning, the mice were housed in groups of six animals in filter topped polysulfone cages 267 × 207 × 140 mm (370 cm^2^ floor area) (Tecniplast, Buguggiate, VA, Italy) using corncob bedding (Scobis Due, Mucedola SRL). Environmental enrichment consisted of soft tissue paper and shredded paper to stimulate the natural behavior of nesting.

### 4.2. Molecular Analysis: Macrodissection, RNA Isolation, cDNA Synthesis, and Real-Time Quantitative PCR Analysis

In order to assess the transcription levels of the *Itpr2* gene, relative mRNA levels of the *Itpr2* gene were quantified by real-time quantitative reverse-transcriptase polymerase chain reaction analysis (qRT-PCR). For this, the WT (*n* = 8), Q135 (*n* = 9), IP_3_R2 KO (*n* = 3), and IP_3_R2 KO; Q135 (*n* = 5) animals were euthanized, their brains were harvested, and their brainstems (a key affected region in SCA3) were macrodissected.

Total RNA was isolated from the tissues using TRIZOL (Invitrogen, Waltham, MA, USA) according to the manufacturer’s protocol. First-strand complementary DNA (cDNA), synthesized using the iScript™ cDNA Synthesis Kit (Bio-Rad, Hercules, FL, USA), was amplified by qRT-PCR according to the guidelines (Bio-Rad, Hercules, FL, USA).

The primers used in this study (Table 1) were designed using PRIMER-BLAST (NCBI, Bethesda, Rockville, ML, USA; http://www.ncbi.nlm.nih.gov/tools/primer-blast/, accessed on 1 June 2019). Quantification was performed using the Fast Real-Time PCR System (Applied Biosystems, Waltham, MA, USA). The housekeeping beta-2-microglobulin (*B2m*) gene was used as an internal control. The relative gene expression was determined using the 2^−ΔΔCt^ relative quantification method and represented as fold change normalized to the mean of the relative expression of the wild-type mice. The PCR cycling conditions used were denaturation at 95 °C for 30 s, 30 cycles at 95 °C for 5 s, annealing at 60 °C for 30 s, plus an extension at 65 °C for 5 s, and a final extension at 95 °C for 5 s.

### 4.3. Behavior Analysis

Behavioral tests were performed during the diurnal period, and the animals were tested at 6, 8, 12, 16, 20, 24, and 30 weeks of age. Body weight was registered at all time points analyzed. Motor behavior was assessed using the balance beam walk test (12-mm square and 11-mm round beams) and the motor swimming test. Furthermore, movement initiation was evaluated with the adhesive removal test, and muscular strength parameters were assessed using the hanging wire grid and the wire maneuver tests. The behavioral tests are briefly explained below:

#### 4.3.1. Motor Swimming Test

The motor swimming test was used to evaluate voluntary locomotion, by taking advantage of the survival instinct of mice in water environments [73]. Each mouse was gently placed at one end of a 100 cm clear acrylic tank filled with water (15 cm depth), maintained at 23 °C ± 1 °C using a thermostat, and trained for 2 consecutive days (3 trials/animal), to reach a visible black platform at the opposite end. During the following 3 days, the animals were tested, and the time that it took each mouse to cross the water tank was recorded (2 trials/mouse) [73]. The tank was labelled with a blue line to mark the starting position, and the mice swam a distance of 60 cm to complete the trial.

#### 4.3.2. Beam Balance Test

Balance and fine motor coordination were evaluated by the latency of each mouse to traverse different diameters and shape beams to reach a safe platform [73]. The beams consisted of long strips of PVC (1 m) with a 12 mm square cross-section or 11 mm round diameter. The beams were placed horizontally, 50 cm above the bench surface, protected with soft sponges to protect the mice from falls, with one end mounted on a narrow support and the other end attached to an enclosed dark box (20 cm square), into which the mouse could escape. The mice were trained for 3 consecutive days on the 12 mm-squared beam (3 trials/animal), and on the fourth day, the animals were tested on both beams (2 trials/animal). The time each mouse took to cross the beams was recorded and discounted if the animal stopped in the beam. A failed trial was considered when an animal fell or turned around on the beam. Each animal had the opportunity to fail twice on each beam [73]. Because the Q135 mice showed a worsening of the phenotype at 16 weeks of age, which affects the ability to perform the task causing them to fall off the beams very frequently, we analyzed the data by attributing performance scores to the animals as follows: 0—performed 2 trials, 1—performed 1 trial, and 2—performed 0 trials, meaning that it cannot walk on the beam.

#### 4.3.3. Adhesive Removal Test

In the adhesive removal test, the experimenter gently placed a round adhesive (8 mm diameter) on the nose of the mouse and transferred the animal to a cage. The time the animal took to remove the adhesive was registered [61,74].

#### 4.3.4. Hanging Wire Grid Test

In the hanging wire grid test, the mice were placed on top of a metallic horizontal grid and inverted 180° towards the surface of the bench (protected with soft sponges to protect the mice from falls). With this test, the latency for the mice to fall off the metal grid was evaluated. The maximum time of the test allowed was 120 s [73,75].

#### 4.3.5. Wire Maneuver Test

The mice were picked up by their tails, and their forelimbs were placed to a fixed wire, underneath of which soft sponges were placed to protect them from falls. This test is based on the latency to fall off the metal wire and the maximum allowed time of the test was 120 s [75].

### 4.4. Statistical Analysis

The G*Power 3.1.9.2 software was used to calculate the required sample size, based on a power of 0.8 (obtained from previous data) [76] and a significance level of 0.05 was used for all statistical tests.

All statistical analyses were performed using SPSS 22.0. The statistical analysis of the behavior tests was performed including all the genotypes under study (including IP_3_R2 −/+); however, the results presented in this study were divided into two groups to simplify the visualizations of the key comparisons as well as to answer the study’s main question. Behavioral data were analyzed by a repeated-measures ANOVA when the variables were continuous or presented a normal distribution. Values that deviated more than 1.5 interquartile ranges from the mean were considered outliers and excluded from further analyses. The assumption of normality was assessed by qualitative analysis of Q-Q plots and frequency distributions (z-score of skewness and kurtosis) as well as by the Kolmogorov–Smirnov and Shapiro–Wilk tests. The assumption of homogeneity of variances was evaluated by Levene’s test. Regarding repeated measurements, sphericity was tested using Mauchly’s test and assumed for all tested variables. For the comparison of means between 5 groups, one-way analysis of variance (ANOVA) was used, followed by Tukey HSD or Dunnett T3’s test (when data passed on the assumption of homogeneity of variances or when the populations variances were not equal, respectively).

Regarding non-normally distributed data and/or for the comparison of medians of discrete variables across time points, a Friedman’s ANOVA was carried out, with pairwise comparisons through the Kruskal–Wallis statistical test.

Effect size measurements are reported for all analyses (Cohen’s d for *t*-tests and eta partial square—n_p_^2^ for ANOVAs). GraphPad Prism 8 was used to create graphs. All figures were created using PowerPoint (version 2305, Microsoft 365 MSO). All statistical information is reported in Appendix A.

## 5. Conclusions

Although behavioral evaluation of the IP_3_R2 KO; Q135 mice revealed the presence of several phenotypic manifestations seen in the CMVMJD135 mouse model, that gradually appear and progress during the mice lifespan, both the Q135 and IP_3_R2 KO; Q135 mice displayed equivalent balance and motor coordination deficits, loss of limb strength, and abnormal gait. Thus, the phenotypical abnormalities induced by mutant human ATXN3 were not altered by the absence of IP_3_R2, the phenotype manifested by the IP_3_R2 KO; Q135 mice which is attributable to the Q135 phenotype already well-described in the literatu [14,71,76,77,78,79,80,81,82,83,84]. This suggests that IP_3_R2 is not a major modulator of disease severity in SCA3.

## Figures and Tables

**Table 1:** Primer sequences used for the analysis of gene expression (qRT-PCR).

Gene	Primer Sequence	
*Itpr1*	Foward	Reverse
	5′-CTCTGTATGCGGAGGGATCTAC-3′	5′-GCGGAGTATCGATTCATAGGAC-3′
*Itpr2*	Foward	Reverse
	5′-CTTCCTCTACATTGGGGACATC-3′	5′-GGCAGAGTATCGATTCATAGGG-3′
*B2m*	Foward	Reverse
	5′-CCTTCAGCAAGGACTGGTCT-3′	5′-TCTCGATCCCAGTAGACGGT-3′

The primers were designed using PRIMER-BLAST (NCBI, http://www.ncbi.nlm.nih.gov/tools/primer-blast/, accessed on 1 June 2019).

## Data Availability

The datasets used and/or analyzed during the current study are available from the corresponding author upon reasonable request.

## Abbreviations

AD: Alzheimer’s disease; ALS: amyotrophic lateral sclerosis; ATXN3: ataxin-3 protein; CNS: central nervous system; ER: endoplasmic reticulum; Het: heterozygous; HD: Huntington’s disease; IP_3_: inositol 1,4,5-trisphosphate molecules; IP_3_Rs: inositol 1,4,5-trisphosphate receptors; IP_3_R2 KO: inositol 1,4,5-trisphosphate receptor type 2 knock-out; IP_3_R2 (−/−): inositol 1,4,5-trisphosphate receptor type 2 knock-out; IP_3_R2 HET: inositol 1,4,5-trisphosphate receptor type 2 heterozygous; IP_3_R2 (+/−): inositol 1,4,5-trisphosphate receptor type 2 heterozygous; KO: knockout; PD: Parkinson’s disease; qRT-PCR: quantitative real-time polymerase chain reaction; RT: room temperature; SCA1: spinocerebellar ataxia type 1; SCA2: spinocerebellar ataxia type 2; SCA3: spinocerebellar ataxia type 3; SCAs: spinocerebellar ataxias; SEM: standard error of the mean; WT: wild-type.

## Appendix A

**Table A1:** Statistical information of behavioral characterization.

***Itpr1***	**One-way ANOVA, with Tukey HSD comparisons** F (3,24) = 0.399, *p* = 0.755 WT vs. Q135 *p* = 0.990 WT vs. IP_3_R2 KO *p* = 0.999 Q135 vs. IP_3_R2; Q135 *p* = 0.706	***n*** (WT) = 8 ***n*** (Q135) = 9 ***n*** (IP_3_R2 KO) = 3 ***n*** (IP_3_R2; Q135) = 5
***Itpr2***	One-way ANOVA, with Dunnett T3 comparisons F (3,24) = 7.888, *p* = 0.001 WT vs. Q135 *p* = 1.000 WT vs. IP_3_R2 KO *p* = 0.027 Q135 vs. IP_3_R2; Q135 *p* < 0.001	*n* (WT) = 8 *n* (Q135) = 9 *n* (IP_3_R2 KO) = 3 *n* (IP_3_R2; Q135) = 5
**CAG** **number**	Independent *t*-test t (15,10) = −0.080, *p* = 0.937	*n* (Q135) = 6 *n* (IP_3_R2; Q135) = 11
**Body Weight**	Repeated Measures ANOVA, with Tukey HSD comparisons Within-subjects effect | F (10,82) = 12.596, *p* < 0.001 n_p_^2^ = 0.604 Between-subjects effect | F (4,33) = 18.178, *p* < 0.001 n_p_^2^ = 0.688 WT vs. Q135 *p* = 0.043 WT vs. IP_3_R2 KO *p* = 0.960 WT vs. IP_3_R2 HET *p* = 0.991 Q135 vs. IP_3_R2; Q135 *p* = 0.008	*n* (WT) = 12 *n* (Q135) = 9 *n* (IP_3_R2 KO) = 10 *n* (IP_3_R2; HET) = 6 *n* (IP_3_R2; Q135) = 12
	One-way ANOVA, with Tukey HSD comparisons Week 12 F (4,48) = 11.075, *p* ≤ 0.001 WT vs. Q135 *p* = 0.005 WT vs. IP_3_R2 KO *p* = 0.734 WT vs. IP_3_R2 HET *p* = 0.790 Q135 vs. IP_3_R2; Q135 *p* = 0.342 Week 16 F (4,48) = 12.052, *p* ≤ 0.001 WT vs. Q135 *p* = 0.018 WT vs. IP_3_R2 KO *p* = 1.000 WT vs. IP_3_R2 HET *p* = 0.986 Q135 vs. IP_3_R2; Q135 *p* = 0.426 Week 20 F (4,47) = 28.619, *p* ≤ 0.001 WT vs. Q135 *p* < 0.001 WT vs. IP_3_R2 KO *p* = 0.950 WT vs. IP_3_R2 HET *p* = 0.996 Q135 vs. IP_3_R2; Q135 *p* = 0.133 Week 24 F (4,47) = 34.103, *p* ≤ 0.001 WT vs. Q135 *p* < 0.001 WT vs. IP_3_R2 KO *p* = 0.894 WT vs. IP_3_R2 HET *p* = 1.000 Q135 vs. IP_3_R2; Q135 *p* = 0.085 Week 30 F (4,41) = 30.343, *p* ≤ 0.001 WT vs. Q135 *p* < 0.001 WT vs. IP_3_R2 KO *p* = 1.000 WT vs. IP_3_R2 HET *p* = 1.000 Q135 vs. IP_3_R2; Q135 *p* = 0.076	
**Motor Swimming test**	Repeated Measures ANOVA, with Tukey HSD comparisons Within-subjects effect | F (15,116) = 5.660, *p* < 0.001 n_p_^2^ = 0.422 Between-subjects effect | F (4,31) = 10.592, *p* < 0.001 n_p_^2^ = 0.577 WT vs. Q135 *p* = 0.003 WT vs. IP_3_R2 KO *p* = 0.960 WT vs. IP_3_R2 HET *p* = 0.982 Q135 vs. IP_3_R2; Q135 *p* = 0.994	*n* (WT) = 12 *n* (Q135) = 9 *n* (IP_3_R2 KO) = 10 *n* (IP_3_R2; HET) = 6 *n* (IP_3_R2; Q135) = 12
	One-way ANOVA, with Tukey HSD comparisons Week 12 F (4,48) = 8.573, *p* ≤ 0.001 WT vs. Q135 *p* = 0.019 WT vs. IP_3_R2 KO *p* = 0.669 WT vs. IP_3_R2 HET *p* = 0.102 Q135 vs. IP_3_R2; Q135 *p* = 0.307 Week 16 F (4,48) = 6.000, *p* = 0.001 WT vs. Q135 *p* = 0.027 WT vs. IP_3_R2 KO *p* = 0.999 WT vs. IP_3_R2 HET *p* = 0.992 Q135 vs. IP_3_R2; Q135 *p* = 1.000 Week 20 F (4,47) = 11.852, *p* ≤ 0.001 WT vs. Q135 *p* = 0.002 WT vs. IP_3_R2 KO *p* = 0.997 WT vs. IP_3_R2 HET *p* = 0.991 Q135 vs. IP_3_R2; Q135 *p* = 0.999 Week 24 F (4,45) = 18,597, *p* ≤ 0.001 WT vs. Q135 *p* < 0.001 WT vs. IP_3_R2 KO *p* = 0.996 WT vs. IP_3_R2 HET *p* = 0.600 Q135 vs. IP_3_R2; Q135 *p* = 1.000 Week 30 F (4,39) = 13,979, *p* ≤ 0.001 WT vs. Q135 *p* < 0.001 WT vs. IP_3_R2 KO *p* = 0.999 WT vs. IP_3_R2 HET *p* = 1.000 Q135 vs. IP_3_R2; Q135 *p* = 0.988	
**Beam** **Walking Test 12 mm square** **(6–20 week)**	Repeated Measures ANOVA, with Tukey HSD comparisons Within-subjects effect | F (10,67) = 4.901, *p* < 0.001 n_p_^2^ = 0.403 Between-subjects effect | F (4,29) = 13.460, *p* < 0.001 n_p_^2^ = 0.650 WT vs. Q135 *p* = 0.028 WT vs. IP_3_R2 KO *p* = 0.784 WT vs. IP_3_R2 HET *p* = 0.986 Q135 vs. IP_3_R2; Q135 *p* = 0.193	*n* (WT) = 12 *n* (Q135) = 9 *n* (IP_3_R2 KO) = 10 *n* (IP_3_R2; HET) = 6 *n* (IP_3_R2; Q135) = 12
	One-way ANOVA, with Tukey HSD comparisons Week 12 F (4,44) = 11.955, *p* < 0.001 WT vs. Q135 *p* = 0.015 WT vs. IP_3_R2 KO *p* = 1.000 WT vs. IP_3_R2 HET *p* = 0.986 Q135 vs. IP_3_R2; Q135 *p* = 0.405 Week 16 F (4,43) = 20.203, *p* < 0.001 WT vs. Q135 *p* = 0.063 WT vs. IP_3_R2 KO *p* = 0.999 WT vs. IP_3_R2 HET *p* = 0.999 Q135 vs. IP_3_R2; Q135 *p* = 0.008 Week 20 F (4,38) = 25.265, *p* < 0.001 WT vs. Q135 *p* < 0.001 WT vs. IP_3_R2 KO *p* = 0.940 WT vs. IP_3_R2 HET *p* = 0.971 Q135 vs. IP_3_R2; Q135 *p* = 1.000	
**Beam Walking Test—12 mm square** **24–30 week** **(Score)**	Kruskal–Wallis test 24 week χ^w^2 (4,47) = 28.174 *p* < 0.001 n_p_^2^ = 0.585 WT vs. Q135 *p* < 0.001 WT vs. IP_3_R2 KO *p* = 1.000 WT vs. IP_3_R2 HET *p* = 1.000 Q135 vs. IP_3_R2; Q135 *p* = 0.972 30 week χ^w^2 (4,40) = 27.513 *p* < 0.001 n_p_^2^ = 0.681 WT vs. Q135 *p* = 0.002 WT vs. IP_3_R2 KO *p* = 1.000 WT vs. IP_3_R2 HET *p* = 1.000 Q135 vs. IP_3_R2; Q135 *p* = 0.743	*n* (WT) = 11 *n* (Q135) = 9 *n* (IP_3_R2 KO) = 10 *n* (IP_3_R2; HET) = 6 *n* (IP_3_R2; Q135) = 11
**Beam Walking Test—11 mm circle** **6–30 week (Score)**	Kruskal–Wallis test 8 week χ^w^2 (4,46) = 16.832 *p* = 0.002 n_p_^2^ = 0.329 WT vs. Q135 *p* = 0.006 WT vs. IP_3_R2 KO *p* = 1.000 WT vs. IP_3_R2 HET *p* = 1.000 Q135 vs. IP_3_R2; Q135 *p* = 0.959 12 week χ^w^2 (4,49) = 29.833 *p* < 0.001 n_p_^2^ = 0.596 WT vs. Q135 *p* = 0.002 WT vs. IP_3_R2 KO *p* = 1.000 WT vs. IP_3_R2 HET *p* = 0.460 Q135 vs. IP_3_R2; Q135 *p* = 0.377 16 week χ^w^2 (4,49) = 36.432 *p* < 0.001 n_p_^2^ = 0.743 WT vs. Q135 *p* < 0.001 WT vs. IP_3_R2 KO *p* = 1.000 WT vs. IP_3_R2 HET *p* = 1.000 Q135 vs. IP_3_R2; Q135 *p* = 0.560 20 week χ^w^2 (4,48) = 29.218 *p* < 0.001 n_p_^2^ = 0.596 WT vs. Q135 *p* = 0.002 WT vs. IP_3_R2 KO *p* = 0.666 WT vs. IP_3_R2 HET *p* = 0.710 Q135 vs. IP_3_R2; Q135 *p* = 0.907 24 week χ^w^2 (4,47) = 38.818 *p* < 0.001 n_p_^2^ = 0.833 WT vs. Q135 *p* < 0.001 WT vs. IP_3_R2 KO *p* = 1.000 WT vs. IP_3_R2 HET *p* = 1.000 Q135 vs. IP_3_R2; Q135 *p* = 0.387 30 week χ^w^2 (4,41) = 26.704 *p* < 0.001 n_p_^2^ = 0.641 WT vs. Q135 *p* = 0.004 WT vs. IP_3_R2 KO *p* = 1.000 WT vs. IP_3_R2 HET *p* = 0.517 Q135 vs. IP_3_R2; Q135 *p* = 0.440	*n* (WT) = 12 *n* (Q135) = 9 *n* (IP_3_R2 KO) = 10 *n* (IP_3_R2; HET) = 6 *n* (IP_3_R2; Q135) = 12
**Adhesive Removal**	Repeated Measures ANOVA, with Tukey HSD comparisons Within-subjects effect | F (10,80) = 4.439, *p* < 0.001 n_p_^2^ = 0.350 Between-subjects effect | F (4,33) = 7.379, *p* < 0.001 n_p_^2^ = 0.472 WT vs. Q135 *p* = 0.097 WT vs. IP_3_R2 KO *p* = 1.000 WT vs. IP_3_R2 HET *p* = 1.000 Q135 vs. IP_3_R2; Q135 *p* = 0.788	*n* (WT) = 12 *n* (Q135) = 9 *n* (IP_3_R2;KO) = 10 *n* (IP_3_R2; HET) = 6 *n* (IP_3_R2;Q135) = 12
	One-way ANOVA, with Tukey HSD comparisons Week 24 F (4,47) = 7.509, *p* < 0.001 WT vs. Q135 *p* = 0.044 WT vs. IP_3_R2 KO *p* = 1.000 WT vs. IP_3_R2 HET *p* = 1.000 Q135 vs. IP_3_R2; Q135 *p* = 0.743 Week 30 F (4,41) = 10.509, *p* < 0.001 WT vs. Q135 *p* = 0.006 WT vs. IP_3_R2 KO *p* = 1.000 WT vs. IP_3_R2 HET *p* = 1.000 Q135 vs. IP_3_R2; Q135 *p* = 0.983	
**Hanging Wire Grid**	Kruskal–Wallis test 6 week χ^w^2 (4,49) = 26.873 *p* < 0.001 n_p_^2^ = 0.531 WT vs. Q135 *p* = 0.018 WT vs. IP_3_R2 KO *p* = 0.855 WT vs. IP_3_R2 HET *p* = 0.637 Q135 vs. IP_3_R2; Q135 *p* = 0.199 8 week χ^w^2 (4,46) = 17,550 *p* = 0.002 n_p_^2^ = 0.346 WT vs. Q135 *p* = 0.006 WT vs. IP_3_R2 KO *p* = 0.700 WT vs. IP_3_R2 HET *p* = 0.761 Q135 vs. IP_3_R2; Q135 *p* = 0.871 12 week χ^w^2 (4,49) = 26.969 *p* < 0.001 n_p_^2^ = 0.533 WT vs. Q135 *p* = 0.009 WT vs. IP_3_R2 KO *p* = 0.678 WT vs. IP_3_R2 HET *p* = 0.718 Q135 vs. IP_3_R2; Q135 *p* = 0.391 16 week χ^w^2 (4,49) = 23.184 *p* < 0.001 n_p_^2^ = 0.449 WT vs. Q135 *p* = 0.031 WT vs. IP_3_R2 KO *p* = 0.799 WT vs. IP_3_R2 HET *p* = 0.813 Q135 vs. IP_3_R2; Q135 *p* = 0.201 20 week χ^w^2 (4,48) = 25.962 *p* < 0.001 n_p_^2^ = 0.522 WT vs. Q135 *p* = 0.006 WT vs. IP_3_R2 KO *p* = 0.675 WT vs. IP_3_R2 HET *p* = 0.920 Q135 vs. IP_3_R2; Q135 *p* = 0.282 24 week χ^w^2 (4,48) = 24.298 *p* < 0.001 n_p_^2^ = 0.484 WT vs. Q135 *p* = 0.008 WT vs. IP_3_R2 KO *p* = 0.587 WT vs. IP_3_R2 HET *p* = 0.459 Q135 vs. IP_3_R2; Q135 *p* = 0.719 30 week χ^w^2 (4,42) = 25.541 *p* < 0.001 n_p_^2^ = 0.593 WT vs. Q135 *p* = 0.014 WT vs. IP_3_R2 KO *p* = 0.699 WT vs. IP_3_R2 HET *p* = 0.883 Q135 vs. IP_3_R2; Q135 *p* = 0.444	*n* (WT) = 12 *n* (Q135) = 9 *n* (IP_3_R2 KO) = 10 *n* (IP_3_R2; HET) = 6 *n* (IP_3_R2; Q135) = 12
**Wire** **Manouver**	Kruskal–Wallis test 6 week χ^w^2 (4,49) = 19.771 *p* = 0.001 n_p_^2^ = 0.373 WT vs. Q135 *p* = 0.011 WT vs. IP_3_R2 KO *p* = 0.210 WT vs. IP_3_R2 HET *p* = 0.560 Q135 vs. IP_3_R2; Q135 *p* = 0.549 12 week χ^w^2 (4,49) = 20.302 *p* < 0.001 n_p_^2^ = 0.384 WT vs. Q135 *p* = 0.020 WT vs. IP_3_R2 KO *p* = 0.976 WT vs. IP_3_R2 HET *p* = 0.616 Q135 vs. IP_3_R2; Q135 *p* = 0.442 16 week χ^w^2 (4,49) = 20.585 *p* < 0.001 n_p_^2^ = 0.391 WT vs. Q135 *p* = 0.004 WT vs. IP_3_R2 KO *p* = 0.699 WT vs. IP_3_R2 HET *p* = 0.568 Q135 vs. IP_3_R2; Q135 *p* = 0.944	*n* (WT) = 12 *n* (Q135) = 9 *n* (IP_3_R2 KO) = 10 *n* (IP_3_R2; HET) = 6 *n* (IP_3_R2; Q135) = 12
